# Two cases of serotypeable and non-serotypeable variants of *Streptococcus pneumoniae* detected simultaneously during invasive disease

**DOI:** 10.1186/s12866-016-0745-0

**Published:** 2016-06-24

**Authors:** Kedibone M. Ndlangisa, Mignon du Plessis, Mushal Allam, Nicole Wolter, Thabo Mohale, Linda de Gouveia, Monica Birkhead, Keith P. Klugman, Anne von Gottberg

**Affiliations:** Centre for Respiratory Diseases and Meningitis (CRDM), National Institute for Communicable Diseases (NICD), a division of the National Health Laboratory Service, Johannesburg, South Africa; School of Pathology, University of the Witwatersrand, Johannesburg, South Africa; Centre for Emerging and Zoonotic Diseases, National Institute for Communicable Diseases (NICD), a division of the National Health Laboratory Service, Johannesburg, South Africa; Hubert Department of Global Health, Rollins School of Public Health, and Division of Infectious Diseases, School of Medicine, Emory University, Atlanta, GA USA

**Keywords:** *wchA*, *cpsE*, Non-serotypeable, Serotype 1, Serotype 18C, Pneumococcus, Mixed culture, South Africa

## Abstract

**Background:**

More than 94 serotypes of *Streptococcus pneumoniae* have been described to date, however the majority of disease is caused by approximately 20 serotypes. Some pneumococci do not react with commercially available antisera used for serotyping and are thus regarded as non-serotypeable (NT). These pneumococci are commonly isolated during carriage studies and very rarely cause invasive disease. Colonization may occur with more than one serotype however disease with more than one serotype is rarely detected. Thus there are limited data describing cases of pneumococcal disease caused by more than one isolate.

**Results:**

In two cases of invasive pneumococcal disease in South Africa, a non-serotypeable and a serotypeable isolate were co-detected during routine serotyping. A serotype 1 and 18C isolate were each co-detected with a non-serotypeable isolate in 2009 (case A) and 2010 (case B), from cerebrospinal fluid and blood, respectively. Both patients were 10–14 years old. For case A, the serotypeable isolate could not be obtained due to low representation in the mixed culture. Using electron microscopy we confirmed lack of capsule for the non-serotypeable isolates. Comparison of the case A non-serotypeable isolate with a serotype 1 genome revealed only the presence of the rhamnose biosynthesis genes (*rmlA*, *B*, *C* and *D*) in the capsular locus, all other capsular genes were absent. Nonetheless it had a multilocus sequence type (ST) associated with serotype 1 (ST217 and ribosomal ST3462) and its core genome clustered with other ST217 isolates. The case B non-serotypeable isolate had all serotype 18C capsular genes except for variation in the *wchA* and *wze* genes, compared to the 18C isolate. Both case B isolates were ST9817 and their core genomes were identical.

**Conclusions:**

The ability of pneumococci to alter capsule production is a potential vaccine escape mechanism and therefore non-serotypeable pneumococci should be monitored as such organisms may increase under vaccine pressure.

**Electronic supplementary material:**

The online version of this article (doi:10.1186/s12866-016-0745-0) contains supplementary material, which is available to authorized users.

## Background

*Streptococcus pneumoniae* (pneumococcus) is a commensal of the human nasopharynx. However, occasionally it evades the immune system and is an important cause of invasive disease such as meningitis and bacteraemia, and non-invasive disease such as pneumonia and acute otitis media. The polysaccharide capsule is known to be a major virulence factor of the pneumococcus. It is immunogenic and can be detected by specific antisera, and hence forms the basis for serotyping and current vaccines against pneumococcal disease. To date, more than 94 serotypes have been described but the majority of disease is caused by approximately 20 serotypes [1]. The most prevalent disease-causing serotypes have been incorporated into currently used polysaccharide conjugate vaccines (PCV-7, -10 and -13). In South Africa, PCV-7 was introduced into the Expanded Programme for Immunisation in 2009 and replaced by PCV-13 in 2011 in a 2 + 1 schedule at 6, 14 and 40 weeks of age [2].

With the exception of serotypes 3 and 37, genes responsible for production of the polysaccharide capsule are located in the capsular polysaccharide (*cps*) region in a single locus flanked by the *dexB* and *aliA* genes [3]. The first four *cps* genes (*cpsA, cpsB, cpsC and cpsD* – also known as *wzg, wzh, wzd and wze*, respectively) are conserved among all serotypes and are involved in the regulation and processing of the capsule [3]. Some pneumococci do not react with commercially available antisera used for serotyping and are thus regarded as non-serotypeable (NT). These pneumococci are commonly isolated during carriage studies and very rarely cause invasive disease [4]. Non-serotypability is due to partial or complete loss of the *cps* gene cluster [5], replacement of *cps* genes with other genes [5–7], sequence duplication [8] or single point mutations, commonly within the *wchA* (also known as *cpsE*) gene [9, 10]. The polysaccharide capsule plays an important role in the pathogenesis of the pneumococcus as it provides protection against complement-mediated phagocytosis. Reduced levels of capsule expression are required for effective attachment to epithelial cells and colonization [11].

While reviewing national surveillance data to identify episodes of mixed invasive pneumococcal disease (IPD) caused by two or more serotypes, we identified two cases (subsequently referred to as case A and case B) whereby one of the co-detected isolates was non-serotypeable (NT). In each of the two cases, the serotypeable and the NT variant was identified from a culture recovered from the same specimen (cerebrospinal fluid for case A and blood for case B). Given the rarity of NT isolates in causing IPD we hypothesized that these NT isolates were variants of their co-detected serotypeable isolates. We therefore compared the genomes of these co-detected NT and serotypeable isolates to determine their relationships.

## Results

Thirty-seven thousand eight hundred twenty-five cases of IPD were reported from 2005 to 2013, of which 70 % (26,475/37,825) had viable isolates. Serotype results were available for nearly all cases with viable isolates (26,469/26,475). A mixed culture with more than one serotype detected (including non-serotypables) was identified for 41 (0.2 %) cases. In two cases, one of the co-detected isolates was NT. These cases were reported in 2009 (case A) and 2010 (case B), respectively and the age range for both was 10–14 year-old (Table 1). These two mixed culture cases were identified during routine serotyping of cultures recovered from cerebrospinal fluid (case A) and blood (case B). Case A was diagnosed with meningitis and clinical information was not available for case B to confirm diagnosis.

**Table 1:** Characteristics of two patients (case A and B) with invasive *Streptococcus pneumoniae* serotypeable and non-serotypeable co-disease, South Africa

	Case A	Case B
Age range in years	10–14	10–14
Gender	male	male
HIV status	unkown	unknown
Specimen	cerebrospinal fluid	blood
Year identified	2009	2010
Diagnosis	meningitis	unknown
Co-detected serotypes	non-serotypeable and serotype 1	non-serotypeable and serotype 18C
Isolated serotypes	^a^non-serotypeable	non-serotypeable and 18C

^a^Pure culture for serotype 1 could not be obtained due to its low representation in the mixed culture (see Results for more information)

For case A, serotype 1 and NT *S. pneumoniae* were identified from the same culture, with the NT isolate representing approximately 98 % of the culture (using the Quellung reaction). The case B isolates were identified as NT and 18C. In each of the two cases, the same results were obtained by two different laboratory staff performing the Quellung reaction on fresh overnight cultures grown from the original transport medium. After several attempts to separate the two variants, only the NT isolate could be obtained for case A, however, real-time PCR confirmed the presence of the serotype 1 *wzy* gene in the mixed culture. For case B, pure cultures were obtained for both the 18C and the NT isolate. The three isolates were susceptible to all tested antimicrobial agents.

**Fig. 1:**
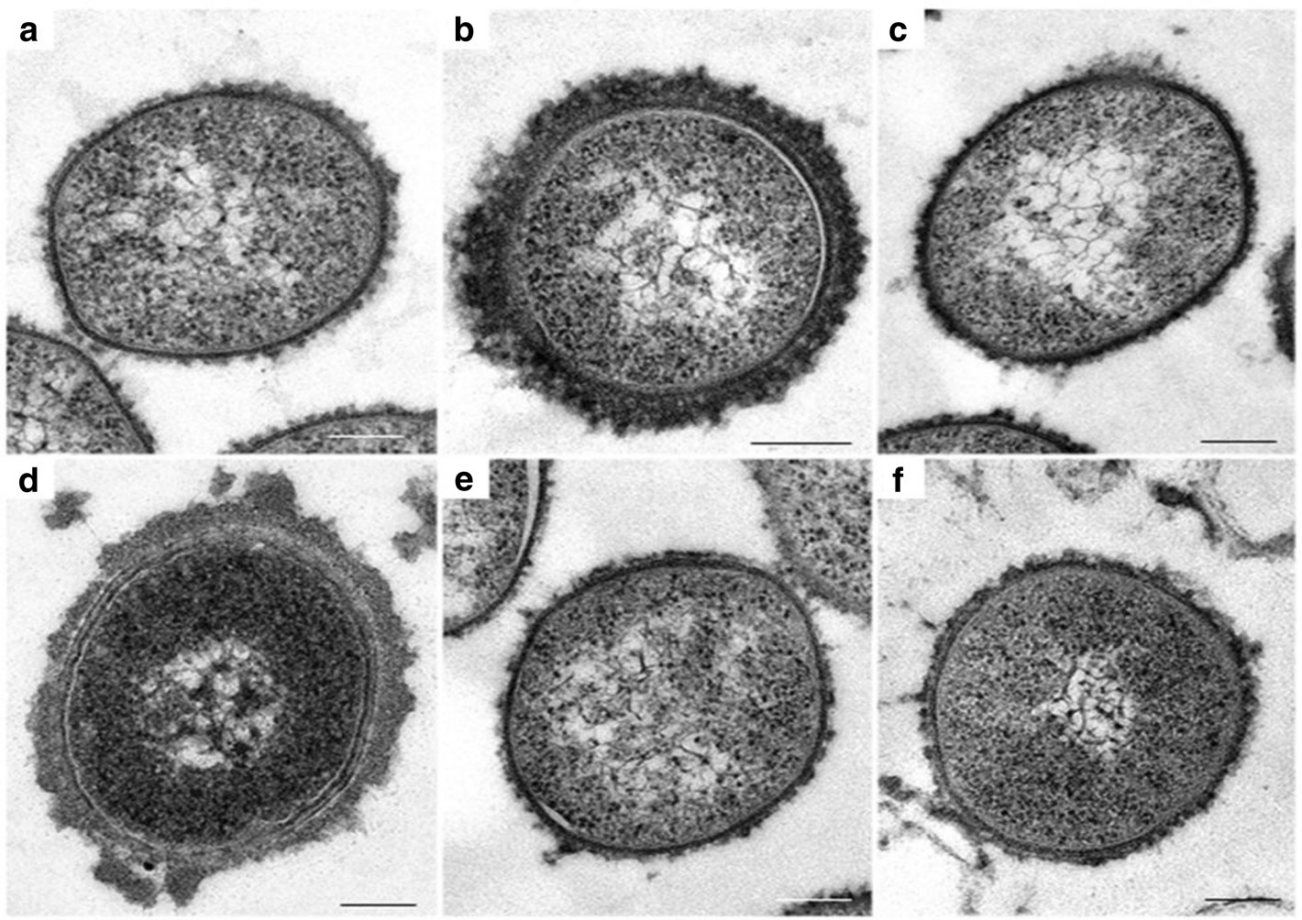
Visualization of pneumococcal isolates using transmission electron microscopy (TEM). Capsular materials of non-serotypable pneumococcal isolates causing mixed infections in two patients in South Africa were compared to capsular materials of serotypeable isolates. The two cases were reported in 2009 (case A) and 2010 (case B). For case A non-serotypeable and a serotype 1 isolate were identified and for case B a non-serotypeable and 18C isolates were identified. TEM of the case A non-serotypeable isolate is shown in **a**, TEM of serotype 1 clinical isolate used as a control in **b**, TEMs of two non-serotypeable clinical isolates used as a controls in **c** and **f**, TEM of case B serotype 18C isolate in **d**, and case B non-serotypeable isolate in **e**. Scale bar = 175 nm

TEM confirmed the absence of capsular material for the case A non-serotypeable isolate (Fig. 1). For case B, 50 to 120 nm thick capsular material was observed around cells of the serotype 18C isolate, while there was no capsular material identified for the NT isolate (Fig. 1).

The genome coverage for the case A NT isolate was 320× and assemblies contained 33 contiguous sequences with a total length of 2,023,465 bp. For the case B NT isolate, the coverage was 355× and assemblies contained 91 contiguous sequences with a total length of 2,110,682 bp. The case B serotype 18C isolate genome coverage was 310× and assemblies contained 78 contiguous sequences with total a length of 2,115,055 bp.

**Fig. 2:**
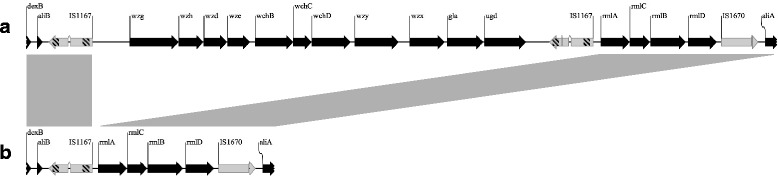
Schematic diagram representing capsular polysaccharide loci of a pneumococcal non-serotypeable and serotype 1 isolate. The schematic diagram represents comparison between the capsular polysaccharide (cps) locus of a non-serotypeable isolate (**b**) co-detected with a serotype 1 isolate during an invasive disease episode in South Africa, 2009, and the cps locus of an invasive serotype 1 clinical isolate from South Africa (**a**). Transposases are indicated by *grey arrows* and flanking repeated sequences by *lines* within the *arrow* delimitations

Sequence analysis of the capsular locus of the case A NT isolate revealed the absence of all but four capsular genes [rhamnose biosynthesis genes (*rmlA*, *B*, *C* and *D*)] (Fig. 2). The isolate was identified as ST217 and rST3462. Because we did not obtain a pure culture for the case A serotype 1 isolate, we compared the case A NT genome sequence to genomes (*n* = 54) of ST217 serotype 1 isolates causing invasive disease in South Africa available on *S. pneumoniae* PubMLST database [12] to further confirm that this NT isolate was indeed a serotype 1 variant. Except for the absence of genes in the capsular region (Additional file 1: Figure S1); the NT core genome was 100 % similar (in 100 % of the bootstrap replications) to sequences of a serotype 1 isolate with the same rMLST profile (rST3462) (Additional file 2: Figure S2). It also clustered with ST217 clinical isolates collected from 1989 to 2013.

For case B, the NT and 18C isolates shared the same new ST (ST9817) and rMLST profile. The NT isolate had all serotype 18C capsule-specific genes [3], however variations were identified in the capsular locus compared to the co-detected 18C isolate and three other genotypically related invasive 18C isolates from South Africa. A nucleotide insertion (472_473 insG) that resulted in a translational frameshift, introducing a stop codon in the *wchA* gene, was identified (Additional file 3: Figure S3). A substitution (149C → A) in the *wze* gene resulted in an amino acid change (T50K) (Additional file 4: Figure S4). Genome sequence comparison of the case B isolates revealed high similarity between the two genomes (Additional file 5: Figure S5) with 100 % identity between their core genomes (Additional file 6: Figure S6).

## Discussion

The polysaccharide capsule is an important component of the pneumococcus that aids in evading the immune system. Hence unencapsulated (non-serotypeable) pneumococci rarely cause invasive disease. Nevertheless, we describe two cases of invasive pneumococcal disease caused simultaneously by what appears to be serotypeable and non-serotypeable variants of the same strain. Our hypothesis, that the NT isolates were variants of their encapsulated counterparts that either lost their capsules or had reduced levels of capsular expression during the infection, is supported by the lack of capsule for both NT isolates from the two cases. Both NT isolates were sequence types of major clones circulating among encapsulated isolates in South Africa [13]. One NT isolate was ST217, the dominant serotype 1 clone in South Africa [13]. The second isolate was a new sequence type (ST9817) that belonged to the ST1016 clonal complex, a predominant clonal complex among serotype 18C isolates in South Africa [13].

Since the NT isolates from both cases were sequence types commonly associated with serotype 1 and 18C, we aligned their *cps* sequences to that of serotype 1 and 18C, respectively. The non-serotypability of the ST217 isolate appears to be caused by the deletion of genes within the *cps* region. This mechanism is common among NT isolates with a serotype 1 genetic background. Scott et al. [14], analysed 28 NT isolates from IPD patients and identified eight isolates with partial deletion of the capsular locus. Four of the eight isolates were a sequence type associated with serotype 1 (ST227) and, similar to our ST217 isolate, only had the rhamnose biosynthesis genes (*rmlA, B, C* and *D*) in their capsular region. Similarly, four IPD NT isolates analysed by Salter et al. [15] that were ST227 only had *rmlA, B, C* and *D* present in their capsular locus. Five additional ST217 NT isolates that were identified among our collection of IPD surveillance isolates and were analysed as part of another study also had only the four rhamnose biosynthesis genes present in the capsular locus (unpublished data). The deletion of the *cps* genes in our ST217 non-serotypeable isolate could be transposon mediated as the deleted serotype 1 genes are flanked by transposase genes (IS1167). We were unable to isolate the encapsulated variant (serotype 1) from this case due to its low representation (approximately 2 %) in the mixed culture. Nevertheless, detection of the serotype 1 *wzy* gene (absent in the genome sequence of the NT isolate) in the mixed culture by real-time PCR supports the presence of serotype 1 in that culture. Our genotypic results also confirm that the NT isolate is a serotype 1 variant as both the sequence type and ribosomal MLST profile are exclusively associated with serotype 1 (ST217 and rST3462). In addition, phylogenetic analysis showed that its core genome clustered with those of other invasive ST217 serotype 1 isolates circulating in South Africa during the same period.

In the second case, the NT isolate had an intact *cps* locus with all serotype 18C capsular genes with the exception of two capsular genes that were variable, compared to the serotype 18C isolate from the same patient. Variation in the *wze* gene resulted in an amino acid change within the protein. Together with *wzd, wze* encodes proteins responsible for the export of the mature polysaccharide to the cell surface [3]. The *wze* gene encodes an auto-phosphorylating protein-tyrosine kinase which is inhibited by aerobic microenvironments, and is thought to be one of the factors causing decreased capsular expression in host environments with higher oxygen levels such as the nasopharynx [11, 16]. Previous studies have demonstrated that mutations in the *wze* gene can inhibit capsule production [11, 16, 17]. An insertion that introduced a premature stop codon in the *wchA* gene was identified in our NT isolate. We suspect that this insertion may be responsible for the loss of capsule expression in our isolate as *wchA* encodes the initial transferase that catalyzes the transfer of glucose-phosphate to the lipid carrier during capsular polysaccharide biosynthesis [18]. Melchior et al. [19] described two mutations in the *wchA* gene (a nucleotide deletion at position 910 and a substitution at position 1081) in two invasive non-serotypeable isolates with serotype 7F capsular genes both of which introduced premature stop codons in the *wchA* gene. Although the positions of these mutations differed from that of our isolate, both resulted in a premature stop codon. In another study, Schaffner et al. [9] recovered a NT isolate, with serotype 18C capsular locus, from the nasopharynx of a child with otitis media. The variations identified in our NT isolate with serotype 18C capsular genes differ from the *wchA* single base substitution (C1135G) described in the Schaffner study.

Repeated sub-culturing in the laboratory may lead to a change in phenotype [20] or the cultures may have become mixed as a result of laboratory error. It is unlikely that this occurred in this instance given the rarity of these cases in our laboratory and the correlating genotypic data between the case B variants. It is favourable for the pneumococcus to have reduced levels of capsule for effective attachment to epithelial cells in the nasopharynx however the capsule is required for survival of the pneumococcus during invasive disease as it provides protection against complement-mediated phagocytosis [11]. We therefore suspect that both variants may have been present in the nasopharynx and invaded simultaneously. Unfortunately we don’t have corresponding nasopharyngeal isolates from these patients to confirm this hypothesis. Another possibility is that the serotypeable variant could have invaded and mutated in the host to lose the capsule. Survival of the NT isolates in the blood and meninges without protection of the capsule is highly irregular but may be related to host factors such as suppressed immune status due to underlying diseases such as HIV infection [21]. Unfortunately we did not have all the relevant patient information to assess if this was true.

## Conclusions

We identified two different vaccine-serotype isolates that appear to have lost their capsules during two separate episodes of invasive disease. Non-serotypeable pneumococci are not targeted by current pneumococcal vaccines and thus present a potential mechanism whereby the organism could escape vaccine pressure. In addition, unencapsulated isolates have higher recombination efficiency than encapsulated pneumococci and therefore provide a reservoir of antibiotic resistance genes for genetic exchange [22]. Antibiotics coupled with routine PCV use may drive the selection of non-serotypeable invasive variants and thus prevalence and genotypes of such pneumococci should be monitored to identify capsular switching or other genetic exchange that may occur under vaccine pressure.

## Methods

### Bacterial strains

Isolates were obtained through Group for Enteric, Respiratory and Meningeal Disease Surveillance (GERMS-SA), a national, laboratory-based surveillance system for IPD in South Africa [23]. Identification of *S. pneumoniae* was based on standardized methodologies using optochin as well as bile solubility on any optochin non-susceptible strains [24]. Once identified, a sweep was inoculated onto Dorset transport medium [Diagnostic Media Products (DMP), Johannesburg, South Africa] and sent to the NICD [25]. Isolates were sub-cultured on arrival at our reference laboratory on 5 % horse blood agar plates (DMP) in the presence of an optochin disc (Mast Group Ltd., United Kingdom). Cultures were stored in 10 % skim milk (DMP) at −70 °C.

### Serotyping and antimicrobial susceptibility testing

Serotypes were determined by the Quellung method using serotype-specific antisera (Statens Serum Institut, Copenhagen, Denmark) [26]. A sweep of culture was initially serotyped and if a mixed culture was suspected, serotyping was repeated on single colonies. Antimicrobial susceptibility testing was performed by the broth microdilution method using commercially prepared Sensititre-SASP2 panels (Trek Diagnostics Inc., Cleveland, OH). Results were interpreted according to Clinical and Laboratory Standards Institute guidelines and breakpoints [27]. The following antimicrobial agents were tested: penicillin G, ceftriaxone, amoxicillin, erythromycin, clindamycin, chloramphenicol, tetracycline, rifampicin, cotrimoxazole, ofloxacin, linezolid and vancomycin. Isolates were considered to be non-susceptible to penicillin at minimum inhibitory concentrations (MICs) ≥0.12 mg/L using the oral penicillin meningitis breakpoints. For other antimicrobials, isolates were defined as non-susceptible if they were intermediately or fully resistant to the agent tested.

### Identification of cases with mixed cultures of a non-serotypeable and a serotypeable isolate

Cases reported for IPD surveillance from 2005 to 2013 were reviewed. A mixed culture was defined as the simultaneous identification of at least two serotypes (including non-serotypeable isolates) either from the same normally-sterile site specimen of an IPD patient or from two or more normally-sterile site specimens obtained from an IPD patient within 21 days of each other. In this study, we characterized isolates from cases where at least one of the co-identified isolates was non-serotypeable. Such cases were detected during routine serotyping by Quellung if an isolate reacted partially with a specific antiserum pool. In such cases, the stored isolate and Dorset transport medium were sub-cultured for single colonies on 5 % horse blood agar plates (DMP). Following 24 h incubation at 37 °C in 5 % CO_2_, the plates were examined for differences in colony morphology and the Quellung reaction was repeated on different colony types. The colonies were sub-cultured, re-identified and serotyped to determine if they were true mixtures. Real-time PCR, targeting the *lytA* gene, was used to confirm non-serotypeable isolates as *S. pneumoniae* [28]. Non-serotypeable isolates were defined as pneumococcal isolates confirmed by *lytA* real-time PCR and for which a serotype could not be assigned by the Quellung method. For the serotypeable isolate and mixed cultures where isolates of different serotypes could not be separated, serotype was confirmed using real-time PCR [29].

### Transmission electron microscopy (TEM)

TEM was conducted to visualize the presence of capsular material. Isolates from overnight cultures were fixed in situ on agar plates, and processed according to the protocol developed by Hammerschmidt et al. with ruthenium red and L-lysine acetate fixation [30]. In addition, three clinical isolates with confirmed serotyping results (one serotype 1 and two NT) were selected from the GERMS-SA collection and were included as controls for TEM analysis.

### Genome sequencing

Overnight fresh cultures were inoculated into brain heart infusion broth (DMP) and incubated overnight at 37 °C in 5 % CO_2_. Cells were pre-lysed at 37 °C for 1 h in 10 mg/ml of lysozyme (Sigma-Aldrich, St. Louis, MO) and DNA was extracted using the QIAamp DNA mini kit (Qiagen, Venlo, Netherlands). DNA extracts were quantified using the Qubit instrument and dsDNA BR Assay kit (Life Technologies, Carlsbad, CA, USA). Multiplexed paired-end libraries were prepared using the Nextera XT DNA sample preparation kit (Illumina, San Diego, CA, USA). Genome sequencing was carried out on an Illumina MiSeq platform. Each isolate was extracted and sequenced three times, each time from a fresh culture.

### Genome analysis

The paired-end reads were checked for quality, trimmed and *de novo* assembled using the Qiagen CLC Genomics Workbench version 8 (Qiagen, Venlo, Netherlands). The resultant contiguous sequences were then ordered using Mauve [31] and *S. pneumonia*e ATCC 700669 as a reference [GenBank:FM211187] and annotated using Prokka version 1.11 [32]. In addition the sequenced reads were mapped against references for capsular regions of co-detected serotypes [GenBank: CR931632 and CR931673].

Variations within the capsular polysaccharide biosynthesis genes were detected using the fixed ploidy variant detection tool within the CLC Genomics Workbench. This model detects the variants whose representation in the reads is in accordance with the assumed ploidy, discards variants whose representation in the reads is likely due to sequencing errors or mapping artefacts and then reports on positions where there may be single nucleotide variation (SNV) including insertions, deletions and substitutions. Identified variations were further investigated using CLC Genomic workbench to identify amino acid changes.

Sequences of the 7 multilocus sequence typing (MLST) genes were extracted from the assembled genomes and allele numbers and sequence types (ST) assigned using the Bio-MLST-Check module (http://search.cpan.org/dist/Bio-MLST-Check/lib/Bio/MLST/Check.pm). Furthermore, contiguous sequences were uploaded to the *S. pneumoniae* PubMLST isolates database [12], which runs the Bacterial Isolate Genome Sequence database (BIGSdb) platform to facilitate the ribosomal MLST (rMLST) analysis [33]. Phylogenetic similarity analysis, based on the core genomes, was performed using the Rapid large-scale prokaryote pan genome analysis (Roary) [34] and the maximum likelihood trees were generated using RaxML version 8 [35]. The genomes of case A and case B isolates were compared to serotype 1 (*n* = 54) and 18C (*n* = 58) genomes randomly selected from sequences available on *S. pneumoniae* PubMLST database [12]. The selected sequences were of serotype 1 and 18C isolates that caused IPD in South Africa from 1989 to 2013 and 2005 to 2013, respectively. For the analysis, core genomes consisted of genes that were present as single copies in at least 90 % of isolates being analysed and, in addition, had the same sequence length in every isolate. The BLAST Ring Image Generator (BRIG) was used to visualise similarities between genomes of mixed culture isolates [36].

## Abbreviations

GERMS-SA, Group for Enteric, Respiratory and Meningeal Disease Surveillance; *S. pneumoniae*, *Streptococcus pneumoniae*; IPD, invasive pneumococcal disease; HIV, human immunodeficiency virus; PCV, polysaccharide conjugate vaccine; CO_2_, carbon dioxide; DMP, Diagnostic media production; MIC, minimum inhibitory concentration; PCR, polymerase chain reaction; NT, non-serotypeable; cps, capsular polysaccharide; TEM, transmission electron microscopy; Roary, Rapid large-scale prokaryote pan genome analysis; MLST, multilocus sequence typing; rMLST, ribosomal multilocus sequence typing; ST, sequence type; BIGSdb, Bacterial Isolate Genome Sequence database; SNV, single nucleotide variation; BRIG, BLAST Ring Image Generator

## Additional files

Additional file 1: Figure S1. BLAST Ring Image of the complete genome sequence of a non-serotypeable and serotype 1 isolate. This figure shows comparison between genome sequence of a non-serotypeable (NT) isolate co-detected with a serotype 1 isolate during a single episode of invasive disease in South Africa in 2009 and genome sequences of a serotype 1 isolate of the same sequence type (ST) 217 and ribosomal ST3462 as the NT isolate. (PDF 191 kb) Additional file 2: Figure S2. Core genome phylogenetic tree of Case A non-serotypeable isolate and serotype 1 isolates from South Africa. The tree represents phylogenetic comparison of the core genome of a non-serotypeable (NT) co-detected with a serotype 1 isolate in 2009 in South Africa and genomes of serotype 1 isolates (*n* = 54) that caused invasive disease in South Africa from 1989 to 2013. This maximum likelihood tree was built using core gene sequences which were concatenated and aligned, using MUSCLE (EMBL-EBI). The numbers at the nodes indicate RAxML bootstrap values. Branch lengths are proportional to the number of substitutions per site (see the scale bars). The colour coding is according to sequence type (ST). (PDF 173 kb) Additional file 3: Figure S3. Comparison of *wchA* gene sequences of 18C and non-serotypeable isolates from a South African patient. Amino acid sequences of *wchA* genes of two isolates [serotype 18C and non-serotypeable (NT)] recovered simultaneously from a patient with invasive pneumococcal disease in South Africa in 2010 were aligned to identify differences between the two sequences. The region where the NT amino acid sequence differs from the 18C sequence is enclosed in a box. Position of a stop codon within the NT amino acid sequence is indicated by an asterisk (*). (PDF 150 kb) Additional file 4: Figure S4. Comparison of *wze* gene sequences of 18C and non-serotypeable isolates from a South African patient. This figure shows amino acid sequence alignment of *wze* genes of two isolates [serotype 18C and non-serotypeable (NT)] recovered from a patient with invasive pneumococcal disease in South Africa is shown. The box indicates a variable amino acid due to a single nucleotide variation between the two isolates. (PDF 177 kb) Additional file 5: Figure S5. BLAST Ring Image (BRIG) of the complete genome sequence of a non-serotypeable and serotype 18C isolate. This figure visualise similarities between genomes of mixed culture isolates. The two isolates [serotype 18C and non-serotypeable (NT)] were co-detected during a single episode of invasive disease in South Africa in 2010. (PDF 197 kb) Additional file 6: Figure S6. Core genome phylogeny of a non-serotypeable isolate and serotype 18C isolates from South Africa. The tree represents phylogenetic comparison of the core genome of two isolates that were co-detected during a single episode of invasive disease in South Africa in 2010 [serotype 18C and non-serotypeable (NT)] and genomes of serotype 18C isolates (*n* = 58) that caused invasive disease in South Africa from 2005 to 2013. This maximum likelihood tree was built using core gene sequences which were concatenated and aligned, using MUSCLE (EMBL-EBI). The numbers at the nodes indicate RAxML bootstrap values. Branch lengths are proportional to the number of substitutions per site (see the scale bars). The colour coding is according to sequence type (ST). (PDF 164 kb)
