# Supplementary figures and images for: Two cases of serotypeable and non-serotypeable variants of Streptococcus pneumoniae detected simultaneously during invasive disease
Source: BMC Microbiol. 2016 Jun 24;16:126. doi: 10.1186/s12866-016-0745-0 (PMC4921036; doi:10.1186/s12866-016-0745-0)

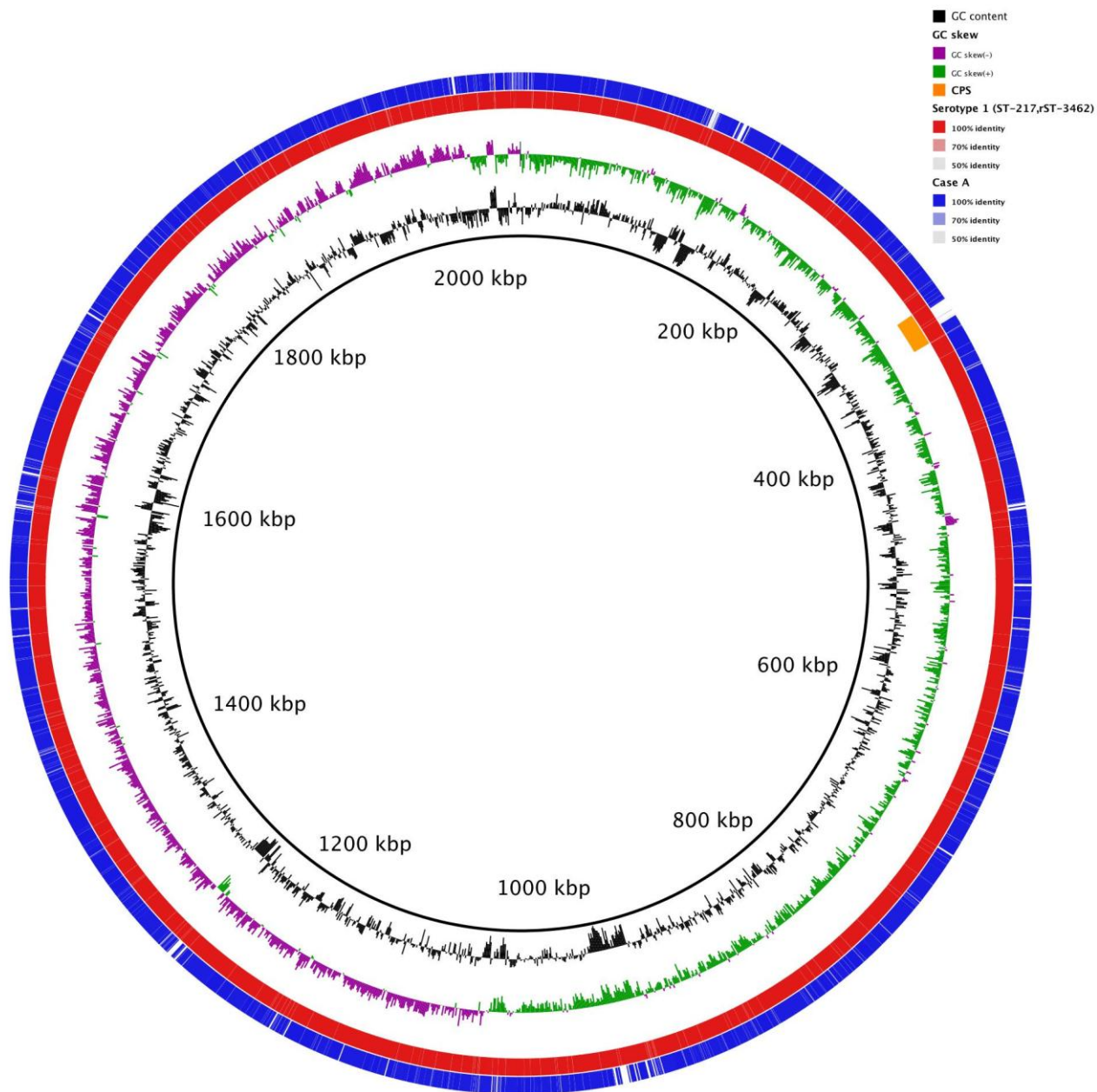

Supplement: Additional file 1: Figure S1. — BLAST Ring Image of the complete genome sequence of a non-serotypeable and serotype 1 isolate. This figure shows comparison between genome sequence of a non-serotypeable (NT) isolate co-detected with a serotype 1 isolate during a single episode of invasive disease in South Africa in 2009 and genome sequences of a serotype 1 isolate of the same sequence type (ST) 217 and ribosomal ST3462 as the NT isolate. (PDF 191 kb) [file 12866_2016_745_MOESM1_ESM.pdf]

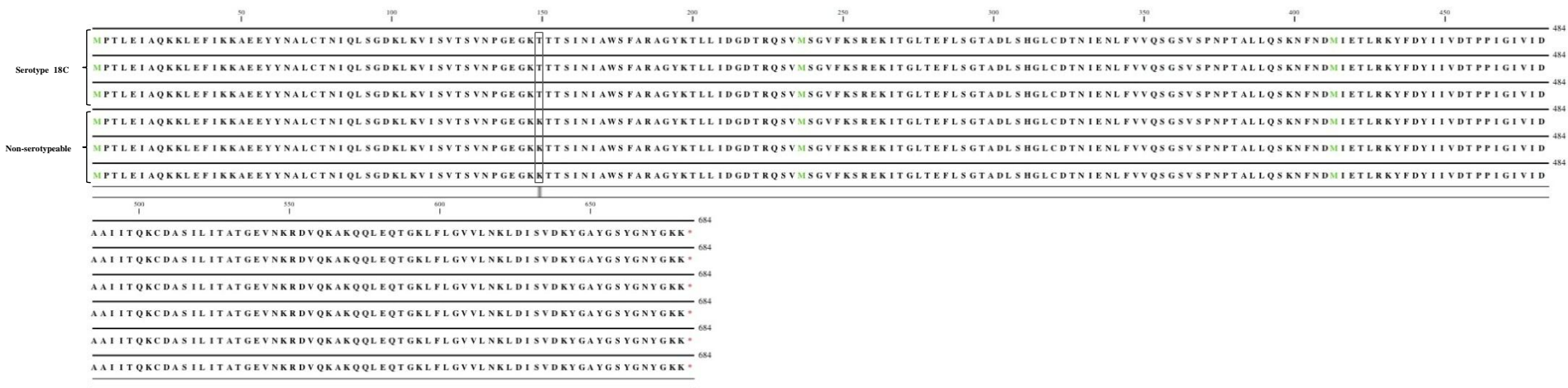

Supplement: Additional file 4: Figure S4. — Comparison of wze gene sequences of 18C and non-serotypeable isolates from a South African patient. This figure shows amino acid sequence alignment of wze genes of two isolates [serotype 18C and non-serotypeable (NT)] recovered from a patient with invasive pneumococcal disease in South Africa is shown. The box indicates a variable amino acid due to a single nucleotide variation between the two isolates. (PDF 177 kb) [file 12866_2016_745_MOESM4_ESM.pdf]

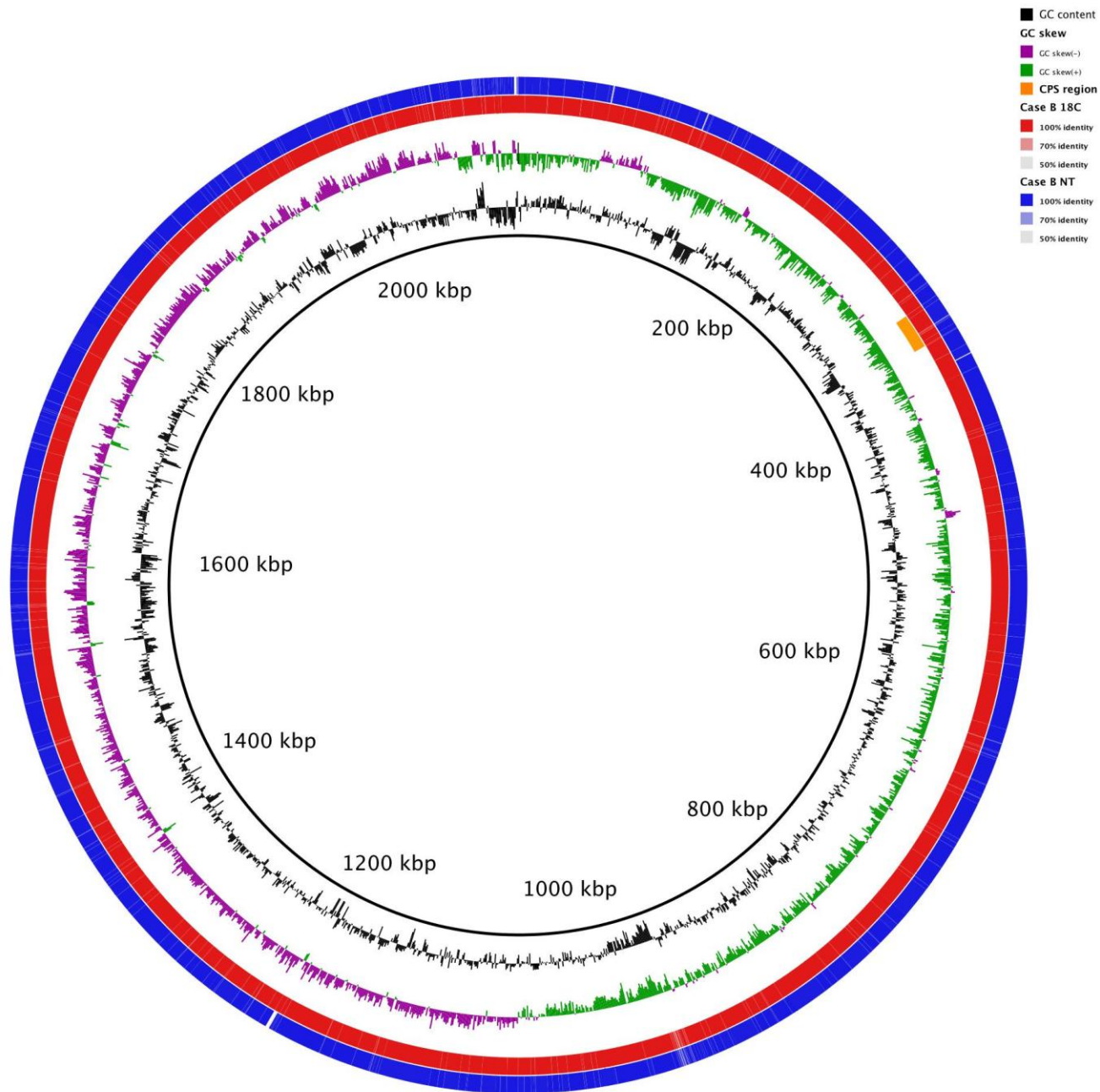

Supplement: Additional file 5: Figure S5. — BLAST Ring Image (BRIG) of the complete genome sequence of a non-serotypeable and serotype 18C isolate. This figure visualise similarities between genomes of mixed culture isolates. The two isolates [serotype 18C and non-serotypeable (NT)] were co-detected during a single episode of invasive disease in South Africa in 2010. (PDF 197 kb) [file 12866_2016_745_MOESM5_ESM.pdf]

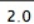

Supplement: Additional file 6: Figure S6. — Core genome phylogeny of a non-serotypeable isolate and serotype 18C isolates from South Africa. The tree represents phylogenetic comparison of the core genome of two isolates that were co-detected during a single episode of invasive disease in South Africa in 2010 [serotype 18C and non-serotypeable (NT)] and genomes of serotype 18C isolates (n = 58) that caused invasive disease in South Africa from 2005 to 2013. This maximum likelihood tree was built using core gene sequences which were concatenated and aligned, using MUSCLE (EMBL-EBI). The numbers at the nodes indicate RAxML bootstrap values. Branch lengths are proportional to the number of substitutions per site (see the scale bars). The colour coding is according to sequence type (ST). (PDF 164 kb) [file 12866_2016_745_MOESM6_ESM.pdf]
