# Supplementary material for: Two cases of serotypeable and non-serotypeable variants of Streptococcus pneumoniae detected simultaneously during invasive disease
Source: BMC Microbiol. 2016 Jun 24;16:126. doi: 10.1186/s12866-016-0745-0 (PMC4921036; doi:10.1186/s12866-016-0745-0)
Supplement: Additional file 3: Figure S3. — Comparison of wchA gene sequences of 18C and non-serotypeable isolates from a South African patient. Amino acid sequences of wchA genes of two isolates [serotype 18C and non-serotypeable (NT)] recovered simultaneously from a patient with invasive pneumococcal disease in South Africa in 2010 were aligned to identify differences between the two sequences. The region where the NT amino acid sequence differs from the 18C sequence is enclosed in a box. Position of a stop codon within the NT amino acid sequence is indicated by an asterisk (*). (PDF 150 kb) [file 12866_2016_745_MOESM3_ESM.pdf]

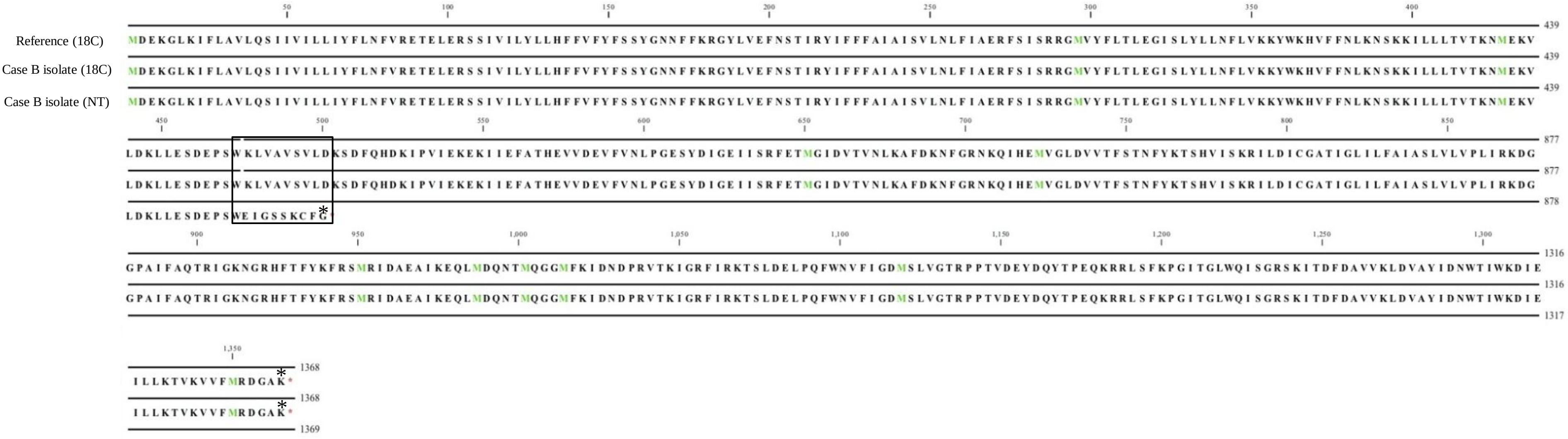

50 100 150 200 250 300 350 400
439
Reference (18C) MDEKGLKIFLAVLQSIIVILLIYFLNFVRETELERSSIVILYLLHFFVFYFSSYGNNFFKRGYLVEFNSTIRYIFFFAIAISVLNLFIAERFSISRRGMVYFLTLEGISLYLLNFLVKKYWKHVFFNLKNSKKILLLTVTKNMEKV
439
Case B isolate (18C) MDEKGLKIFLAVLQSIIVILLIYFLNFVRETELERSSIVILYLLHFFVFYFSSYGNNFFKRGYLVEFNSTIRYIFFFAIAISVLNLFIAERFSISRRGMVYFLTLEGISLYLLNFLVKKYWKHVFFNLKNSKKILLLTVTKNMEKV
439
Case B isolate (NT) MDEKGLKIFLAVLQSIIVILLIYFLNFVRETELERSSIVILYLLHFFVFYFSSYGNNFFKRGYLVEFNSTIRYIFFFAIAISVLNLFIAERFSISRRGMVYFLTLEGISLYLLNFLVKKYWKHVFFNLKNSKKILLLTVTKNMEKV
450 500 550 600 650 700 750 800 850
877
LDKLLESDEPSWKLVAVSVLDKSDFQHDKIPVIEKEKIIEFATHEVVDEVFVNLPGESYDIGEIISRFETMGIDVTVNLKAFDKNFGRNKQIHEMVGLDVVTFSTNFYKTSHVISKRILDICGATIGLILFAIASLVLVPLIRKDG
877
LDKLLESDEPSWKLVAVSVLDKSDFQHDKIPVIEKEKIIEFATHEVVDEVFVNLPGESYDIGEIISRFETMGIDVTVNLKAFDKNFGRNKQIHEMVGLDVVTFSTNFYKTSHVISKRILDICGATIGLILFAIASLVLVPLIRKDG
878
LDKLLESDEPSWEIGSSKCFG*
900 950 1,000 1,050 1,100 1,150 1,200 1,250 1,300
1316
GPAIFAQTRIGKNGRHFTFYKFRSMRIDAEAIKEQLMDQNTMQGGMFKIDNDPRVTKIGRFIRKTSLDELPQFWNVFIGDMSLVGTRPPTVDEYDQYTPEQKRRLSFKPGITGLWQISGRSKITDFDAVVKLDVAYIDNWTIWKDIE
1316
GPAIFAQTRIGKNGRHFTFYKFRSMRIDAEAIKEQLMDQNTMQGGMFKIDNDPRVTKIGRFIRKTSLDELPQFWNVFIGDMSLVGTRPPTVDEYDQYTPEQKRRLSFKPGITGLWQISGRSKITDFDAVVKLDVAYIDNWTIWKDIE
1317
1,350
1368
ILLKTVKVVFMRDGAK*
1368
ILLKTVKVVFMRDGAK*
1369
