# Supplementary material for: Two cases of serotypeable and non-serotypeable variants of Streptococcus pneumoniae detected simultaneously during invasive disease
Source: BMC Microbiol. 2016 Jun 24;16:126. doi: 10.1186/s12866-016-0745-0 (PMC4921036; doi:10.1186/s12866-016-0745-0)
Supplement: Additional file 2: Figure S2. — Core genome phylogenetic tree of Case A non-serotypeable isolate and serotype 1 isolates from South Africa. The tree represents phylogenetic comparison of the core genome of a non-serotypeable (NT) co-detected with a serotype 1 isolate in 2009 in South Africa and genomes of serotype 1 isolates (n = 54) that caused invasive disease in South Africa from 1989 to 2013. This maximum likelihood tree was built using core gene sequences which were concatenated and aligned, using MUSCLE (EMBL-EBI). The numbers at the nodes indicate RAxML bootstrap values. Branch lengths are proportional to the number of substitutions per site (see the scale bars). The colour coding is according to sequence type (ST). (PDF 173 kb) [file 12866_2016_745_MOESM2_ESM.pdf]

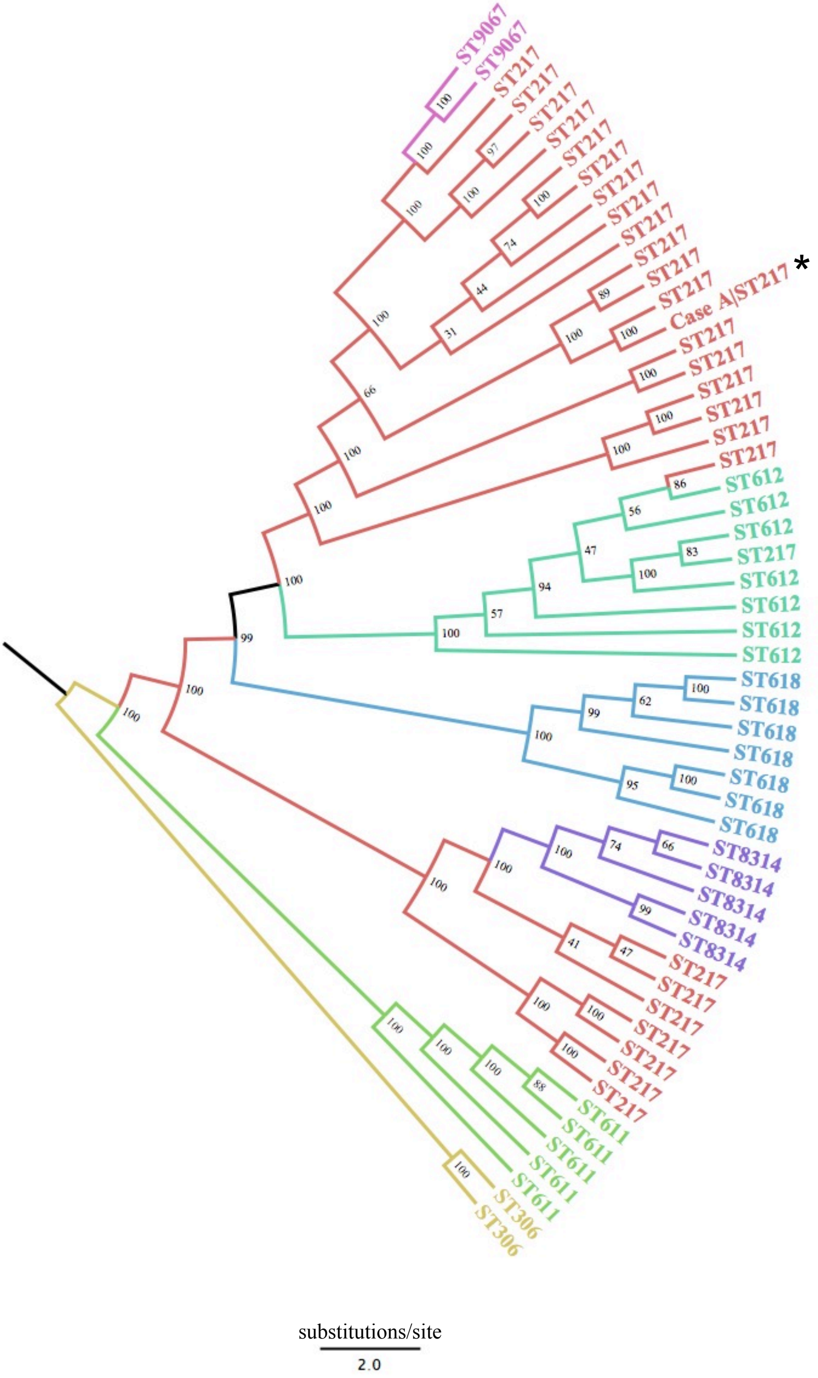
ST9067
ST9067
ST217
ST217
ST217
ST217
ST217
ST217
ST217
ST217
ST217
ST217
ST217
Case A|ST217 *
ST217
ST217
ST217
ST217
ST217
ST217
ST612
ST612
ST612
ST217
ST612
ST612
ST612
ST612
ST618
ST618
ST618
ST618
ST618
ST618
ST618
ST8314
ST8314
ST8314
ST8314
ST8314
ST217
ST217
ST217
ST217
ST217
ST217
ST611
ST611
ST611
ST611
ST611
ST306
ST306
substitutions/site
2.0
